# Reduced ErbB4 Expression in Immune Cells of Patients with Relapsing Remitting Multiple Sclerosis

**DOI:** 10.1155/2011/561262

**Published:** 2011-09-12

**Authors:** Evgenia Tynyakov-Samra, Eitan Auriel, Yifat Levy-Amir, Arnon Karni

**Affiliations:** Neuroimmunology laboratory, Department of Neurology, Tel Aviv Sourasky Medical Center, Sackler's Faculty of Medicine, Tel Aviv University, 64239 Tel Aviv, Israel

## Abstract

*Background.* There is an insufficient remyelination in the lesions of multiple sclerosis (MS). One of the factor that was found to promote remyelination is neuregulin-1 which is the ligand of ErbB4. Immune cells have been implicated in neurogenesis and oligodendrogenesis. *Aims.* We studied the expression of ErbB4 in the immune cells of patients with relapsing remitting (RR) multiple sclerosis (MS) and healthy controls. *Methods.* ErB4 expression in immune cells was studied by flow cytometry without stimulation or with stimulation with anti-CD3 and anti-CD28 monoclonal antibodies or in the presence of interferon-g or TNF-**α** as well as by immunoprecipitation and Western blot, and its mRNA was studied by real-time PCR. *Results.* We found reduced levels of ErbB4 in the total PBMCs and in T cells, monocytes, and B cells of RR MS patients. Similarly, the ErbB4 RNA levels were reduced in the immune cells of patients with RR-MS. Stimulation via CD3 and CD28 significantly upregulated the expression of ErbB4 on immune cells healthy individuals. This effect was weaker in the patients group. *Conclusion.* ErbB4 may play a role in the proliferation of oligodendrocyte progenitor cells, differentiation of oligodendrocytes, and remyelination, and, therefore, the reduced ErbB4 expression in immune cells of patients with RR-MS may contribute to insufficient remyelination that occurs in the disease.

## 1. Introduction

Multiple sclerosis (MS) is a chronic inflammatory demyelinating disease of the central nervous system that is responsible for the most common cause of neurological disability in young adults [1]. MS plaques are characterized by the presence of immune cells infiltration, demyelination, death of mature oligodendrocytes axonal damage, and neurodegeneration [2]. Neuronal precursor cells (NPCs) and oligodendrocyte precursor cells (OPCs) are present MS lesions [3], and the process of remyelination exists in the lesions of MS [4, 5]; however, this process is mostly insufficient and fail to remyelinate successfully. Neuregulins are a family of ligands that exert trophic effects on both neurons and glia via their receptors ErbB2, ErbB3, and ErbB4. It was shown that a soluble isoform of neuregulin-1, glial growth factor 2(GGF2), promotes survival and proliferation of glial cells and their progenitors and enhances remyelination in vivo [6–8]. ErbB4 has been shown to participate in wide spectrum of functions and to take a crucial role in the development of the nervous system and the heart as well as in diseases like cancer and schizophrenia [9–12]. Moreover, an upregulated expression of ErbB4 was seen on surviving oligodendrocytes and on reactive microglial cells in and around MS lesions, where myelin and oligodendrocyte depletion occur and was found to be expressed on lymphocytes in lymph nodes [13]. It was recently reported that neuregulin-1 is involved in immune regulation [14]. 

It has long been suggested that the immune system may have a role in assisting the repair and regeneration of the central nervous system (CNS) damaged tissue by myelin-reactive T cells and T cell-derived cytokines [15, 16] by specifically activated blood-borne myeloid cells [17–19].

In view of the potential role of ErbB4 expression in myelin regeneration in MS and neuroregenerative potential of the immune activity, the aim of the present study was to investigate the expression profile of ErbB4 in immune cells of patients with MS. 

## 2. Methods

Patients with MS attending the Neuroimmunology Clinic at the Tel Aviv Sourasky Medical Center were included in the study. After the participants had given their informed consent, blood samples were drawn from 13 patients with definite relapsing remitting MS (RR-MS) according to revised McDonald et al. criteria and 10 aged-matched healthy controls (HC) (Table 1). Peripheral blood mononuclear cells (PBMCs) were isolated from venous blood samples by centrifugation over Ficoll-Paque (Amersham biosciences Uppsala, Sweden). ErbB4 receptor expression in PBMCs was studied by flow cytometry using phycoerythrin (PE) conjugated mouse monoclonal antibodies (mAb) against CD3, CD14, and CD19 (R&D Systems) as well as intracellular staining for ErbB4 with mouse and human ErbB4 mAb (Santa Cruz) and allophycocyanin-(APC-)conjugated F(ab)_2_ against human Fc (Jackson ImmunoReasearch) and the appropriate isotype controls. In a further experiment, PBMCs of 5 MS patients and 5 HC were cultured for 24 hrs with either anti-CD3 mAb and anti CD28 mAb (R&D Systems) or with the corresponding isotype controls for 24 hrs or in the presence of interferon-*γ* 100 ng/mL or TNF-*α* 100 ng/mL for 24 hrs. The detection of these molecules was done by FACScan flow cytometer (Beckton Dickinson). The analysis was done by CellQuest Software (Beckton Dickinson) for the measurement of the specific mean fluorescence intensity (MFI) of ErbB4 on the detected cells and the percentages of ErbB4 positive cells. 

In addition ErbB4 expression in PBMCs was studied by immunoprecipitation and Western blot analyses. Large cell carcinoma H661 cell line with high expression of ErbB4 was used as positive control, while H1299 human lung adenocarcinoma cell line was used as negative control. PBMCs were extracted using buffer RIPA. Insoluble material was removed by 15 min centrifugation (12,000 × g) at 4°C. Supernatants (0.5 mg protein) were incubated for 2 h at 4°C with monoclonal ErbB4 antibody, followed by additional incubation for 1 h at 4°C with protein G Agarose beads. Immunocomplexes were washed twice with buffer PBS-Tween and once with PBS. The beads were suspended in Laemmli's Sample buffer, boiled for 5 min, resolved by means of 10% SDS-PAGE, and were immunoblotted with anti-ErB-4 polyclonal antibody. During the immunoprecipitation, immediately after extraction, we determined the protein level of all samples using Bradford reagent, after we incubate similar amounts of total proteins with the anti-ErbB4 antibody and further incubate with bead, we loaded the same amount of supernatant. Therefore, we assume that the same amounts of supernatant were loaded on the gel. 

Total RNA was prepared from PBMCs using an Easy RNA purification kit (Biological Industries) according to the manufacturer's instructions. The total RNA samples were routinely treated with Turbo-DNase (Ambion) to prevent possible genomic DNA contamination. Before the real-time qRT-PCR reactions studied, the absence of residual genomic DNA contamination were tested using PCR reaction with GAPDH primers of samples that did not undergo in vitro transcription reaction. The total RNA of 1 *μ*g was transcribed with random hexamers using Reverse iT transcriptase (Verso cDNA kit) following the manufacturer's instructions. Real-time qRT-PCR was performed on a ABI Prism 7900 HT Instrument (Applied Bioscience). ErbB4 mRNA expression was tested by qRT-PCR performed as follows: 94°C for 10 minutes and 45 cycles as following: 94°C for 15 seconds, 60°C for 15 seconds, and 72°C for 15 seconds. The melting curve analysis was routinely used for each reaction. The GAPDH gene was run in parallel for internal control for each reaction set. The GAPDH gene was run in parallel for internal control for each reaction set. The uniformity of GAPDH expression in PBMCs was tested by GeneVestigator program, analyzing several different microarrays studies. GAPDH expression was independent of any PBMC treatment in healthy individuals and in both groups of RR-MS patients as was described before [20].The oligonucleotide primer sequences used were ErbB4 forward primer: 5′-GGC TGC TGA GTT TTC AAG GAT G-3′ and ErbB4 reverser primer 5′-GCT TCA TAC GAT CAT CAC CCT GA-3′, GAPDH forward primer 5′-ACCACAGTCCATGCCATCAC-3′ and GAPDH reverse primer 5′ TCCACCACCTGTTGCTGTA-3.

The data presented for RNA expression is a calculated relative quantification values according ABI PRISM 7900 HT software and normalized against GAPDH ± S.E.

All the data are presented as mean ± S.E. Statistical analyses for comparing the ErbB4 expression levels between the study groups were carried out by Student's *t*-test.

## 3. Results

**Figure 1:**
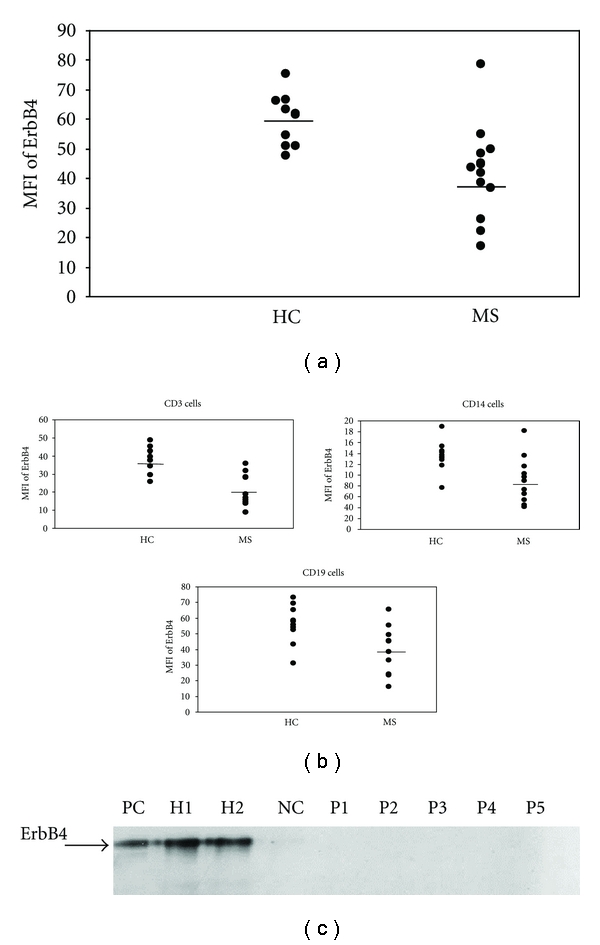
ErbB4 expression in unstimulated PBMCs of patients with RR-MS and aged matched healthy controls as was measured by flow cytometry. The mean fluorescence intensity (MFI) of ErbB4 in PBMCs of patients with RR-MS was significantly lower than that in PBMCs of healthy controls (a). The MFI of ErbB4 on unstimulated T cells, monocytes, and B cells of patients with RR-MS was significantly reduced as compared with healthy controls (b). After immunoprecipitation with monoclonal ErbB4 antibody, the cell lysates were processed by Western blot analysis and probed by polyclonal antibody. Large cell carcinoma H661 cell line was used as positive control (PC). H1299 human lung adenocarcinoma cell line was used as negative control (NC). The donors here are different from those that are described in figure a and b: H1-2 are healthy controls, and P1-5 are for RR-MS patients (c).

We first compared the ErbB4 expression in PBMCs of patients with RR-MS and age-matched HC by flow cytometry and found that the mean fluorescence intensity (MFI) of ErbB4 in PBMCs of patients with RR-MS was significantly lower (37.8 ± 2.8) than in HC (60.0 ± 5.1, *P* = 0.002) (Figure 1(a)). However, no significant differences were found in the comparison of the percentages of ErbB4-positive PBMCs (39.7 ± 1.3% in the RR-MS versus 41.3 ± 1.4 in the HC, *P* = 0.61). 

We thereafter examined the expression of ErbB4 in the different cell types among PBMCs (T cells = CD3^+^ cells, monocytes = CD14^+^ cells and B cells = CD19^+^ cells) in the study groups (Figure 1(b)). We found that the MFI of ErbB4 expression in T cells was lower in RR-MS patients (22.2 ± 3.3) than in HC (37.5 ± 2.4, *P* = 0.002), the MFI of ErbB4 expression in monocytes was lower in RR-MS patients (88.5 ± 11.5) as compared to HC (135.7 ± 8.9, *P* = 0.006), and the ErbB4 expression in B cells was lower in RR-MS patients (39.8 ± 4.9) as compared to HC (56.2 ± 3.9, *P* = 0.018). Again similar percentages of ErbB4, positive cells subtypes were found between the patient group and the HC group. An average of 54.2 ± 2.0% of the T cells in the patients versus 57.0 ± 2.1% in HC, 16.5 ± 1.5% of the monocytes in the patients versus 15.0 ± 1.5% in HC and 7.7 ± 0.9 of the B cells in the patients versus 8.3 ± 0.9 in HC. All these comparisons of percentage of positive cells were statistically insignificant. 

In order to confirm our observations, we also studied the expression of the ErbB4 protein by immunoprecipitation and western blotting. Similarly, ErbB4 was detected at the expected molecular weight range (180 kDa) in PBMCs of 2 HC but not in PBMCs of 5 RR-MS patients (Figure 1(c)).

**Figure 2:**
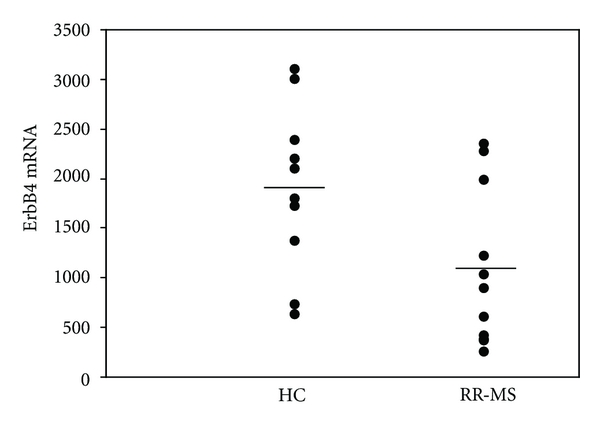
The levels of ErbB4 mRNA expression in PBMCs were studied by real-time relative RT-PCR in 10 RR-MS and 10 age- and sex-matched healthy controls. Normalization of ErbB4 mRNA expression was done with regards to the GAPDH mRNA levels of expression.

In order to further explore the differences of ErbB4 expression between our study groups, we studied the mRNA level of expression of ErbB4 by real-time PCR and found significant lower relatives expression of ErbB4 mRNA in RR-MS patients (1067.0 ± 239.0) as compared to HC (1903.1 ± 265.3, *P* = 0.030) (Figure 2).

**Figure 3:**
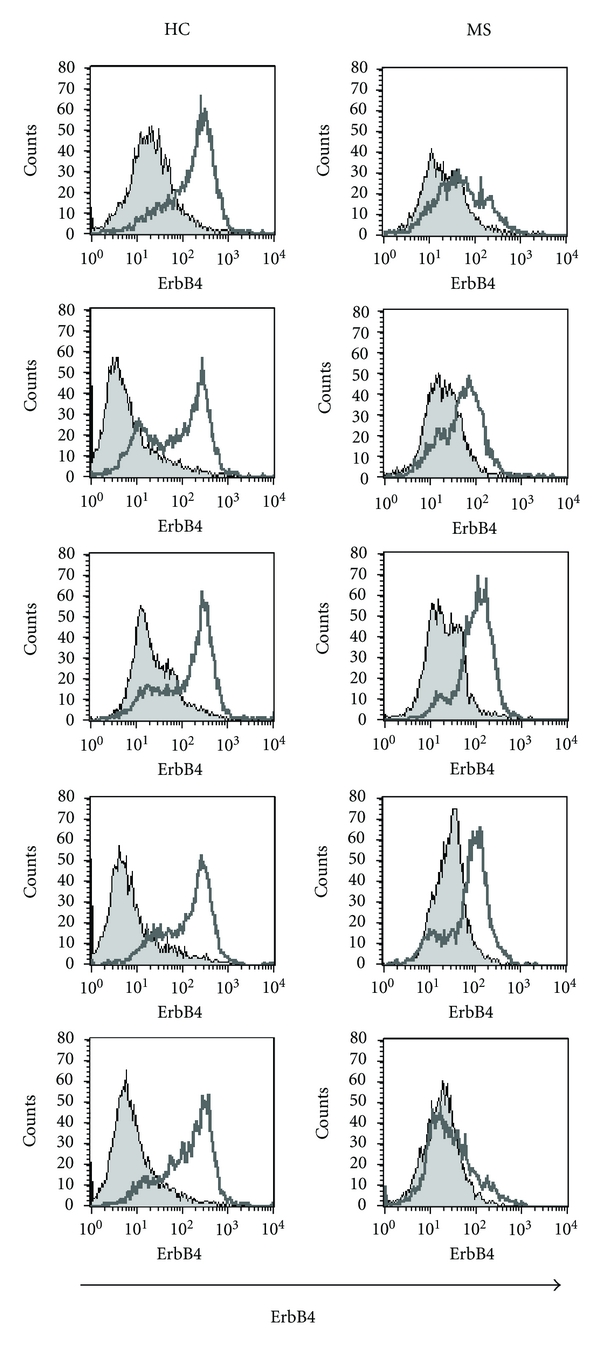
ErbB4 expression in PBMCs of 5 patients with RR-MS and 5 age- and sex-matched healthy controls (HC) was studied after 24 hrs incubation with anti-CD3/CD28 mAb (white histograms) or their isotype controls (grey histograms). The stimulatory effect via CD3/CD28 on the MFI of ErbB4 was significantly higher in the HC as compared to the patients.

We further studied the stimulatory effect of anti-CD3/CD28 mAb on the expression of ErbB4 on the PBMCs in a different set of participants (Figure 3). Stimulation with anti CD3/CD28 mAb upregulated the expression of ErbB4 to a significantly lesser extent in the MS patients group, the average of the ratios of ErbB4 MFI between with anti-CD3/CD28 mAb and isotype controls (average ratio ± S.D = 3.5 ± 1.6) as compared to HC (6.2 ± 1.6, *P* = 0.005). In both patients group and HC group, there was an upregulation of the ErbB4 after stimulation with anti-CD3/CD28 mAb, but this effect in the patients group (60.8 ± 18.4) did not reach the levels of MFI in the HC (106.4 ± 18.8, *P* = 0.04). No differences were found in the percentages of PBMCs positive ErbB4 between MS patients and HC after stimulation with anti-CD3/CD28 mAb (average percentages for MS patients ± S.D = 13.2 ± 9.7, *P* = 0.038 and in HC = 13.0 ± 6.0, *P* < 0.001). No significant effect was found on the ErbB4 MFI after 24 hrs incubation with interferon-*γ* between the patients group (the MFI average ratio versus without cytokine = 1.5 ± 1.1) and HC (1.2. ± 0.6, *P* = 0.591) or on the percentages of ErbB4-positive PBMCs (the percentages average ratio versus without cytokine ± S.D = 20.7 ± 39.8 *P* = 0.370) and in HC (9.9 ± 16.5, *P* = 0.386). Similarly, no significant effect was found on the ErbB4 MFI after 24 hrs incubation with TNF-*α* between the patients group (the MFI average ratio versus without cytokine = 1.1 ± 0.6) and HC (0.7 ± 0.3, *P* = 0.240) or on the percentages of ErbB4-positive PBMCs (the percentages average ratio versus without cytokine of ErbB4 + PBMCs in the patients group (10.9 ± 20.8, *P* = 430) and in the HC (1.69 ± 1.8, *P* = 438). We did not find differences between untreated patients and patients that were treated with interferon-b with regards to the expression of ErbB4.

## 4. Discussion

Neuregulins have been shown in to support several types of glial cells by enhancing their proliferation, survival, and differentiation as well as the enhancement of remyelination in the adult brain [6]. It is important to note, that the process of remyelination is mostly inconsistent in MS lesions, and many lesions fail to remyelinate successfully. Furthermore, in part of the patients the MS lesion is mostly characterized by oligodendrocyte apoptosis [2]. This failure contributes to irreversible axon loss and progressive neurological deterioration [21, 22].

In the present study, we looked for the expression of the natural receptor of neuregulins, ErbB4, and found for the first time that ErbB4 levels are reduced in the PBMCs of patients with RR-MS. This reduced expression was found in all cell subset we studies: T cells, monocytes, and B cells and was also demonstrated in the transcripts levels of ErbB4 mRNA. The stimulation via CD3/CD28 upregulated the expression of ErbB4 in HC and had a weaker effect in the patients with MS. We assume that ErbB4 may play a role in the proliferation of oligodendrocyte progenitor cells and in the differentiation of oligodendrocytes, and therefore, an inadequate ErbB4 expression may be related to insufficient remyelination that occurs in the disease. Our results disclose a new unreported aspect that is related to the deviated immunity in MS that includes the increased production of proinflammatory cytokines, such as IFN-*γ* from T cells and IL-12, IL-18 and IL-23 from monocytes and dendritic cells [23–27], the loss of function of Treg cells, such as CD4^+^CD25^+^ T cells and CD46-mediated Tr-1 cells [28, 29], as well as of suppressor CD8+ T cells [30, 31] and the reduction in immune-mediated neurotrophins, and noggin production [20, 32, 33]. In MS, there is an increased proinflammatory activity and a presumed decreased immune-mediated regulatory, neuroprotective, and neuroregenerative activity. We suggest that the reduced expression and responsiveness to CD3/CD28 stimulation of ErbB4 expression on immune cells in patients with MS may be related to an insufficient immune mediation of remyelination and oligodendrogenesis in MS.

## Figures and Tables

**Table 1:** Study Participants.

Participants	Blood donors (*n*)	Age (yrs)	Female: male
All RR MS patients	18	34.7 ± 14.2	12 : 6
Untreated patients	7	32.1 ± 8.5	5 : 2
Interferon-*β*-treated patients	11	36.2 ± 9.9	7 : 4
Healthy controls	15	35.6 ± 6.8	10 : 5
